# Supplementary material for: Factors affecting sustainable adoption of e-health technology in developing countries: an exploratory survey of Nigerian hospitals from the perspective of healthcare professionals
Source: PeerJ. 2018 Mar 1;6:e4436. doi: 10.7717/peerj.4436 (PMC5835346; doi:10.7717/peerj.4436)
Supplement: Supplemental Information 1 [file peerj-06-4436-s001.pdf]

APPENDIX

Questionnaire Items For E-health Survey

Please indicate your opinion on each of the item with a tick "√" based on scale 1-7:
1=strongly disagree, 2=disagree, 3=slightly disagree, 4=neutral, 5=slightly agree, 6=agree, 7=strongly agree.

**Perceived Usefulness**	**1**	**2**	**3**	**4**	**5**	**6**	**7**
Using e-health system enables me to accomplish tasks more quickly							
Using e-health system increases my work productivity							
Using e-health system enhances my effectiveness on the job							
Using e-health system makes it easier to do my work							
**Attitude towards e-health**	**1**	**2**	**3**	**4**	**5**	**6**	**7**
Using e-health system gives me greater control over my work							
Using e-health system improves my work performance							
Using e-health system saves me time to conduct my work							
Using e-health system improves the quality of the work I do							
**Intention to use e-health**	**1**	**2**	**3**	**4**	**5**	**6**	**7**
Using e-health system is usually frustrating							
The e-health system is rigid and inflexible to use							
I find it cumbersome to use the e-health system							
I find the e-health system useful in my job							
I find the e-health system easy to use							
**Staff IT experience**	**1**	**2**	**3**	**4**	**5**	**6**	**7**
I have sufficient knowledge of the e-health system							
I am aware of the potential benefits of the e-health system							
The hospital have IT experts to train staff in using the e-health system							
**Information sharing**	**1**	**2**	**3**	**4**	**5**	**6**	**7**
The e-health system enhances information sharing within the hospital							
The e-health system enhances information sharing with other hospitals							
The hospitals' policies and regulations do not affect the use of the e-health system							
Overemphasis on protecting patients' privacy affects the use of the e-health system							
**Security concerns**	**1**	**2**	**3**	**4**	**5**	**6**	**7**
There are security measures to protect information and preserve confidentiality							
There are security measures to communicate accurate information							
There are security measures to protect medical records from unauthorized access							
Overemphasis on security affects the effective use of e-health system							
**Technical infrastructures**	**1**	**2**	**3**	**4**	**5**	**6**	**7**
There are available computers to support the use of e-health system							
The hospitals' IT budget is enough to sustain the e-health system							
The hospitals' existing infrastructure is enough to support the e-health system							
There is a system in place to maintain the hospitals' existing infrastructure							

Demographic questions

Gender: Male ☐ Female ☐

Profession: Doctor ☐ Nurse ☐ Other ☐

Experience: 1-5 ☐ 6-10 ☐ 11-25 ☐

IT knowledge: None ☐ Minimum ☐ Fairly ☐ Maximum ☐

Thank you for participating in this survey!
